# Shikonin Suppresses Human T Lymphocyte Activation through Inhibition of IKK****β**** Activity and JNK Phosphorylation

**DOI:** 10.1155/2013/379536

**Published:** 2013-05-16

**Authors:** Ting Li, Fenggen Yan, Rui Wang, Hua Zhou, Liang Liu

**Affiliations:** State Key Laboratory of Quality Research in Chinese Medicine, Macau Institute for Applied Research in Medicine and Health, Macau University of Science and Technology, Avenida Wai Long, Taipa, Macau

## Abstract

The key role of T cells has been elaborated in mediating immune responses and pathogenesis of human inflammatory and autoimmune conditions. In the current study the effect of shikonin, a compound isolated from a medicinal plant, on inhibition of T-cell activation was firstly examined by using primary human T lymphocytes isolated from buffy coat. Results showed that shikonin dose dependently suppressed T-cell proliferation, IL-2 and IFN-**γ** secretion, CD69 and CD25 expression, as well as cell cycle arrest activated by costimulation of PMA/ionomycin or OKT-3/CD28 monoclonal antibodies. Moreover, these inhibitory responses mediated by shikonin were found to be associated with suppression of the NF-**κ**B signaling pathway via inhibition of the IKK**α**/**β** phosphorylation, I**κ**B-**α** phosphorylation and degradation, and NF-**κ**B nuclear translocation by directly decreasing IKK**β** activity. Moreover, shikonin suppressed JNK phosphorylation in the MAPKs pathway of T cells. In this connection, we conclude that shikonin could suppress T lymphocyte activation through suppressing IKK**β** activity and JNK signaling, which suggests that shikonin is valuable for further investigation as a potential immunosuppressive agent.

## 1. Introduction

**Figure 1:**
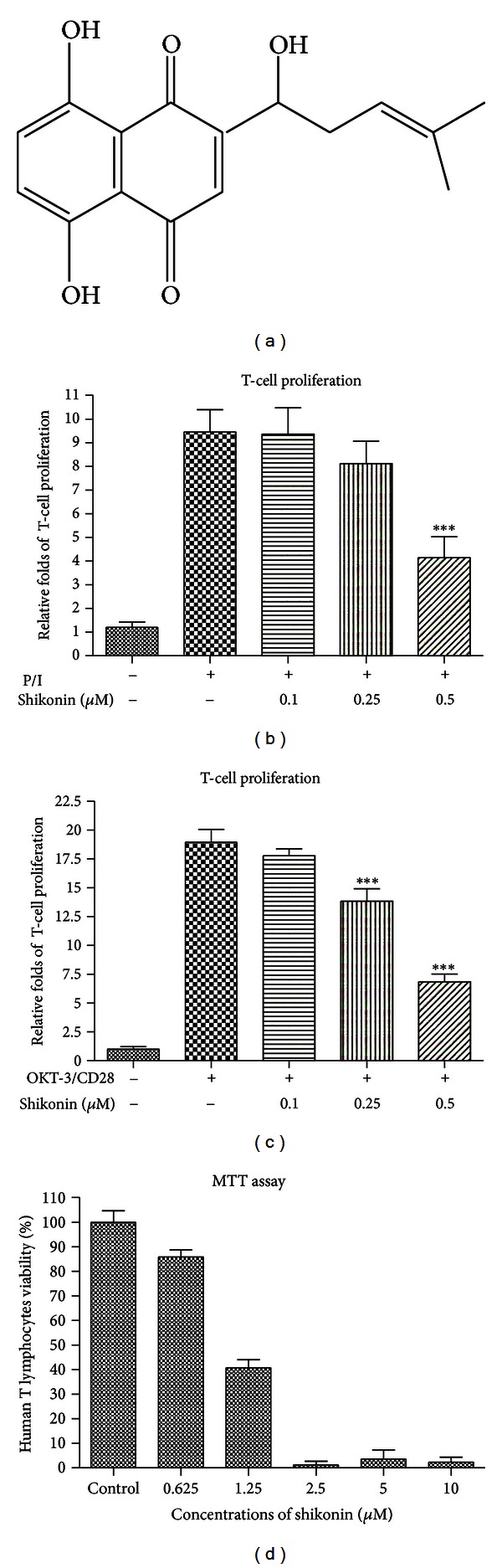
Effect of shikonin on suppression of cell proliferation and its cytotoxicity in human T lymphocytes. Chemical structure of shikonin (a). Effect of shikonin on T lymphocytes proliferation stimulated by PMA/ionomycin (b) or OKT-3/CD28 (c). Human T cells (10^5^/well) were pretreated with the indicated concentrations of shikonin for 2 h and then activated with PMA (20 ng/mL)/ionomycin (1 *μ*M) (P/I) or with the coated OKT-3 (5 *μ*g/mL)/CD28 (1 *μ*g/mL) (OKT-3/CD28) for 72 h. BrdU was added to the cells for 14 h incubation before the end of cell culture, and then the amount of BrdU incorporation was measured by using plate reader at 450 nm. Data are expressed as relative folds of BrdU incorporation of the controlled cells and represent the mean ± SEM of three independent experiments. Cytotoxicity of shikonin on human T lymphocytes (d). The cells (10^5^/well) were treated with shikonin at the indicated concentrations for 3 days, and then MTT reagent was added to the cells for 4 h of incubation followed by addition of solubilization buffer. The absorbance was then read at 570 nm. Data are expressed as the percentage of absorbance of controlled cells and represent the mean ± SEM of three independent experiments.

The red naphthoquinone pigment shikonin (Figure 1(a)) is the major bioactive component in the roots of *Lithospermum erythrorhizon* Sieb. et Zucc. (Boraginaceae), which possesses a number of medical properties like relieving measles, macular eruptions, sore throat, burns, and carbuncles. According to the theories of Chinese and Korean traditional medicine, it is believed to possess properties of removing heat from the blood and detoxification [1] and claimed to be beneficial for burns anal ulcers, haemorrhoids, infected crusts, bedsores, external wounds, and oozing dermatitis [2]. It was also reported to have anti-inflammatory, antithrombotic, and antitumor action [3–5]. These effects were produced by inhibition of proteasome in primary macrophages, downregulation of NF-*κ*B/MAPK activation [6], prevention of NF-*κ*B to DNA in RAW264.7 cell line [7], suppression of gene expression of TNF-*α*, IL-1*β* and IL-4, chemokines CCL4 and CCL8, as well as the inflammatory modulators NFATC3 and PTGS2 [8]. In addition, shikonin showed to inhibit maturation of bone marrow-derived dendritic cells *in vitro* [2]. However, there is no report about the action and mechanism of shikonin on T cells, a dominant cell population for mediating immune and inflammatory responses in humans.

NF-*κ*B is a ubiquitous and well-characterized transcriptional factor in cellular signaling during T-cell activation, which regulates a large number of genes involving immune, inflammatory, and antiapoptotic responses [9]. In resting T cells, NF-*κ*B is bound to I*κ*B*α* in cytoplasm, existing as a heterodimer composed by p65 and p50 proteins. When T cells are activated by stimuli, I*κ*B kinase (IKK) and two site-specific critical serine residues of I*κ*B*α* (Ser32, Ser36) are phosphorylated. Subsequently, the phosphorylation form of I*κ*B*α* is hence ubiquitinated, cleaved by the 26S proteasome, and then degraded. Hence then NF-*κ*B is released and translocated into the nucleus of cells, where it binds to *κ*B enhancer element of DNA, and induces transcription of many inflammatory mediators [10], and finally leads to activation of T cells. Therefore, due to the key role of NF-*κ*B signaling in regulating T-cell activation and immune response, it is one of the important strategies to develop NF-*κ*B signaling for drug discovery in the past decade [11–13]. Although NF-*κ*B activity can be suppressed by inhibition of 26S proteasome, IKK activity, or interfering with binding of NF-*κ*B to DNA, IKK*β* activity has been evident of playing the pivotal role in regulating NF-*κ*B activation. As such, screening selective IKK*β* inhibitors would be an effective strategy for developing anti-inflammatory therapeutics [13].

In addition, the mitogen-activated protein kinases (MAPKs), a family of serine/threonine, have been known as the central pathway of T-cell activation and one of the most attractive targets for intervening inflammatory and autoimmune conditions. MAPKs contain the signature sequence -TXY-, where T and Y are threonine and tyrosine, and X is glutamate, proline, or glycine, in ERK, JNK, or p38, respectively [14]. To date, four components of MAPKs have been identified, that is, the extracellular signal-regulated kinases (ERK1/2), c-Jun NH2-terminal kinase (JNK-1/2/3), p38 (p38*α*/*β*/*γ*/*δ*), and ERK5. Among them, p38 and JNK can be activated by cellular stresses, called as stress-activated MAPKs. Taken together, both NF-*κ*B and MAPKs are the major signaling pathways involving T-cell activation and the attractive targets for developing anti-inflammation and immunomodulation drugs.

Shikonin has been previously reported effectively for anti-inflammation, antithrombosis and antitumor through downregulation of NF-*κ*B/MAPK activation in primary macrophages, while the effect of shikonin on human T-cell activation has never been reported. In the current study we demonstrated the action of shikonin on the cell proliferation, cell cycle, expression of cell surface activation marker, and modulation of NF-*κ*B and MAPKs signaling in human T lymphocytes.

## 2. Materials and Methods

### 2.1. Drugs and Reagents

Shikonin of >98% purity verified by HPLC was obtained from Merck & Co. (Rahway, NJ, USA). Pan T-Cell Isolation Kit II was purchased from MACs (Earhart Avenue, CA, USA). Anti-human Phycoerythrin (PE)-CD3 antibody and other antibodies of fluorescein isothiocyanate (FITC)-CD25, FITC-CD69, FITC-CD71, NF-*κ*B, and OKT3 antibody were from BD Pharmingen Inc. (San Diego, CA, USA). CD28 monoclonal antibody was purchased from eBioscience (San Diego, CA, USA). Phorbol 12-myristate 13-acetate (PMA) and ionomycin were obtained from Sigma and Calbiochem, respectively. FLAG-tagged IKK*β* wildtype (wt) was gift from Tom Gilmore (Boston University) and checked by standard DNA sequencing. The primary antibodies used in the current study were rabbit antibodies specific for I*κ*B*α*, IKK-*α*/*β*, p-IKK*α*/*β*, and p-I*κ*B*α* ^ser32^ (Cell signaling, USA), mouse antibodies specific for actin (Santa Cruz, USA). Both IL-2 and IFN-*γ* ELISA kit were purchased from Invitrogen (Carlsbad, CA, USA).

### 2.2. Human T-Cell Isolation, Purification, and Stimulation

Human peripheral blood T lymphocytes were isolated from buffy coat blood, based on the method described previously [15]. Briefly, the buffy coat blood obtained from Macau blood transfusion center was mixed with normal saline and then transferred to Ficoll-Paque (Amersham Biosciences, USA) in 50 mL tubes. The mixture was centrifuged at 350 g for 35 min to separate the blood into layers. The layer of mononuclear cells was collected, and then all of cells were purified by MACs pan T-cell kit. Human T lymphocytes were cultured in RPMI 1640 medium supplemented with 10% fetal bovine serum (FBS). To stimulate T lymphocyte activation, two sets of costimulators, that is, 20 ng/mL PMA plus 1 *μ*M ionomycin (PMA/ionomycin) or immobilized 5 *μ*g/mL OKT-3 antibody plus 1 *μ*g/mL CD28 antibody (OKT-3/CD28), were used. According to the different purposes of the experiments, one set of costimulators from the above two was employed in each experiment, with different time intervals of stimulation and cell culture.

### 2.3. T Lymphocyte Proliferation and Cytotoxicity Assay

T lymphocyte proliferation assay was conducted by cell proliferation kit (Roche, USA) according to the manufacturer's instruction. Briefly, 100 *μ*L human T lymphocytes (1 × 10^6^/mL) were cultured in 96-well plates in triplicate in 1640 medium plus 10% FBS. The cells were then stimulated with 20 ng/mL PMA plus 1 *μ*M ionomycin or coated 5 *μ*g/mL OKT-3 plus 1 *μ*g/mL CD-28 in the presence or absence of shikonin for 72 h. BrdU was added to the cells at final concentration of 10 *μ*M and then following incubated for another 14 h. BrdU can incorporate into the dividing cells in their DNA; thus, quantification of BrdU incorporation shows the degree of cell proliferation. In our current experiments, BrdU was determined by ELISA method, and data were obtained from three independent experiments.

MTT (3-(4,5-dimethylthiazol-2-yl)-2,5-diphenyl tetrazolium bromide) was employed to determine the cytotoxicity as described previously [16]. Briefly, 100 *μ*L human T lymphocytes (1 × 10^5^/well) were cultured in triplicate in a 96-well plate in RPMI 1640 medium plus 10% FBS for 72 h. MTT (5 mg/mL) was added for 4 h incubation, and then a solvent (10% sodium dodecyl sulfate (SDS), 50% N,N-dimethyl formamide, pH 7.2) was added to dissolve the purple precipitate. *A*_570 nm_  was determined from each well on the next day. The percentage of cell viability was calculated using the following formula: Cell viability (%) = *A*_treated_/*A*_control_ × 100. Data reported represent three independent experiments.

### 2.4. Enzyme-Linked Immunosorbent Assay

The level of IL-2 and IFN-*γ* secreted by the activated human T lymphocytes was evaluated by using IL-2 and IFN-*γ* human enzyme-linked immunosorbent assay (ELISA) method (Invitrogen, USA). In brief, cells (1 × 10^5^/well) were incubated in the presence or absence of shikonin for 2 h at different concentrations, and then the cells were stimulated with 5 *μ*g/mL OKT-3 plus 1 *μ*g/mL CD28 or 20 ng/mL PMA plus 1 *μ*M ionomycin for another 48 h. The culture supernatants were collected, and then concentration of IL-2 in the supernatants was determined by ELISA method according to the manufacturer's instructions. All samples were determined in triplicate. Data were obtained from three independent experiments.

### 2.5. T Lymphocyte Surface Marker, Intercellular Protein, and Cell Cycle Analysis

Flow cytometry was employed to evaluate the expressions of T lymphocyte surface markers, including CD25, CD69, and CD71, according to the previously described method [17]. Human T lymphocytes (1 × 10^6^/well) were pretreated with shikonin for 2 h and then stimulated with PMA (20 ng/mL) plus ionomycin (1 *μ*M) [17]. For determination of CD69 expression, the cells were stimulated for 24 h by PMA plus ionomycin; for determination of the expressions of CD25 and CD71 the cells were cultured with stimulators and shikonin for 48 h. At the end of cultures, the cells were harvested and washed with PBS. Cells were then incubated with specific antibodies in the combination of anti-CD69-FITC and anti-CD3-PE, anti-CD25-FITC and anti-CD3-PE, or anti-CD71-FITC and anti-CD3-PE (BD Pharmingen, San Diego, CA, USA), stained for 30 min at room temperature in the dark, and then fixed with 4% PFA paraformaldehyde. On the following day, samples were analyzed on FACS Calibur Flow Cytometer using CellQuest software (BD Biosciences, San Diego, CA, USA). The compensation standards were composed of the separate tubes of cells stained with positive single-color antibodies for each of the fluorochromes.

For analysis of intercellular NF-*κ*B expression using flow cytometry, the cells were incubated with shikonin for 2 h, and then fixed immediately by cytofix buffer after the stimulated by PMA plus ionomycin; subsequently the cells were harvested followed by permeabilization, incubated on ice for 30 min, washed by PBS for three times, and then resuspended in stain buffer containing NF-*κ*B antibody and incubated for 60 min avoiding light. Finally, the cells were washed by stain buffer and analyzed by flow cytometer.

For analysis of cell cycle, human T lymphocytes (10^6^) were treated with shikonin for 2 h and then cultured with or without PMA (20 ng/mL) plus ionomycin (1 *μ*M) for 72 h. After the culture, cells were harvested by centrifugation, washed by PBS, fixed by 70% ethanol, and stained by PI (Propidium Iodide, BD Pharmingen, San Diego, CA, USA) for 30 min at room temperature, and then the cell cycle analysis was measured as the previously reported method after the cells were washed by PBS for three times [18].

### 2.6. Analyses of Cellular Protein Expressions by Using Western Blotting

For detection of I*κ*B*α*, phosphorylation forms of IKK*α*/*β*, total IKK*α*/*β*, phosphorylation forms of JNK (P-JNK), total JNK, phosphorylation forms of ERK1/2 (P-ERK1/2), total ERK1/2, phosphorylation forms of p38 (P-p38) and total p38 kinase from whole cellular proteins, the human T lymphocytes (4 × 10^6^/well) were preincubated with different concentrations of shikonin for 60 min. In determining the phosphorylation form of I*κ*B*α*, the human T lymphocytes (4 × 10^6^/well) were preincubated with different concentrations of shikonin together with 100 *μ*g/mL N-acetyl-leucyl-leucyl-norleucinal (ALLN) (Calbiochem, USA) for 60 min. The cells were then incubated with PMA (20 ng/mL) plus ionomycin (1 *μ*M) for another 60 min and finally harvested. The harvested T lymphocytes were lysed with lysis buffer (50 mM Tris-HCl, pH 7.5, 250 mM NaCl, 5 mM EDTA, 1 mM DTT, 1% Triton, 50 mM NaF, 1 mM sodium orthovanadate, 0.5 mM PMSF and 1× protease inhibitor mix (Roche, USA) to produce whole cellular proteins. The whole cellular proteins were then subjected to electrophoresis in 10% SDS/PAGE and to immunoblotting as mentioned above. The primary antibodies used in this study were rabbit antibodies specific for I*κ*B*α*, P-I*κ*B*α* ^ser32^, IKK*α*/*β* and P-IKK*α*/*β*, P-JNK (Thr183/Try185), JNK, P-ERK1/2 (Thr220/Try204), ERK, P-p38 (Thr180/Try182), p38 (Cell Signaling, USA), and mouse antibodies specific for actin (Santa Cruz, USA).

### 2.7. Transfection and Immunoprecipitation

The transfection assay was conducted according to the manual of lipofectamine LTX (Invitrogen, USA). Briefly, on the day before transfection, trypsinize and count the HEK293T cells, 5 × 10^5^ cells per well were seeded in 1.5 mL of complete DMEM growth medium. For each well of cells to be transfected, 1.25 *μ*g of FLAG-IKK*β* wt plasmid was diluted in 500 *μ*L of Opti-MEM Reduced Serum Media without serum. For each well of cells, 1.25 *μ*L of PLUS was added into the above diluted Opti-MEM : DNA solution, mixed gently, and incubated for 5 min at room temperature. Subsequently, lipofectamine LTX Reagent was added into the above solution and then mixed gently and incubated 30 minutes at room temperature to form DNA-lipofectamine LTX Reagent complexes. After 30 minute incubation, 500 *μ*L of the DNA-lipofectamine LTX Reagent complexes was directly added to each well containing cells and mixed gently. The cells were incubated at 37°C in a CO_2_ incubator for 24 h after transfection.

IKK*β* recombinant protein was pull down by using Flag tagged protein immunoprecipitation Kit (Sigma) according to the manual. In brief, after transfection with Flag-IKK*β* wt for 24 h, HEK293T cells were collected and washed by PBS for twice. The cell lysates were prepared by incubation with lysis buffer for 15 min on ice and then centrifuged for 10 min at 12,000 ×g. The resin was prepared according to the manual, and the cell lysates were added to the resin and agitated for overnight at 4°C. The resin was collected by centrifuging for 30 sec at 8200 ×g and then washed by wash buffer for 3 times. Finally, the Flag-IKK*β* wt was eluted by competition with 3× Flag peptide and stored in −80°C for conducting IKK*β* kinase assay.

### 2.8. IKK Kinase Assay

To determine the direct effect of shikonin on IKK*β* activity, the IKK*β* kinase assay was performed. In brief, both GST-I*κ*B-*α* substrate, FLAG-IKK-*β* wt recombinant protein, and ATP were incubated with or without shikonin at 30°C for 30 min. The mixture was analyzed by 10% SDS-polyacrylamide gel electrophoresis (SDS-PAGE) and then electrotransferred onto nitrocellulose membranes. The nitrocellulose membranes were blocked by 5% dried milk for 60 min and then incubated with P-I*κ*B*α* for overnight at 4°C. Next day, the membranes were washed with TBS-T again and further incubated with HRP-conjugated secondary antibodies for 60 min. The blots were developed using ECL Western Blotting Detection Reagents (Amersham Biosciences).

### 2.9. Statistical Analysis

Data are expressed as means ± SEM. One-way ANOVA or unpaired Student's *t*-test was used to determine the significance of difference; a value of *P* < 0.05 was considered statistically significant.

## 3. Results

### 3.1. Shikonin Inhibits Human T Lymphocyte Proliferation

Optimal T lymphocyte proliferation requires two signals, one is provided by the antigen-specific T-cell receptor (TCR) complex and the other is the costimulatory receptor CD28. In the current study, the immobilized OKT3 (5 *μ*g/mL) plus CD28 (1 *μ*g/mL) antibodies in 96-well plates or PMA (20 ng/mL) plus ionomycin (1 *μ*M) were employed to activate T cells, and the hallmarks of the cell activation could be observed, namely, cell proliferation and secretion of IL-2 and IFN-*γ*. Therefore, we firstly examined the effect of shikonin on human T-cell proliferation, and the results showed that shikonin could suppress the T-cell proliferation induced by OKT-3/CD28 or PMA/ionomycin in a dose-dependent manner (Figures 1(b) and 1(c)). To determine whether the suppressive effect of shikonin on human T lymphocyte proliferation is resulted from the cytotoxicity of the compound, MTT method was employed to evaluate the viability of T cell in the experiment. As shown in Figure 1(d), there is no significant difference on the cell viability between shikonin-treated and nontreated cells at 0.625 *μ*M, so that 0.5 *μ*M shikonin was used as high concentration for further study.

### 3.2. Shikonin Inhibits IL-2 and IFN-*γ* Secretion in Human T Lymphocytes

**Figure 2:**
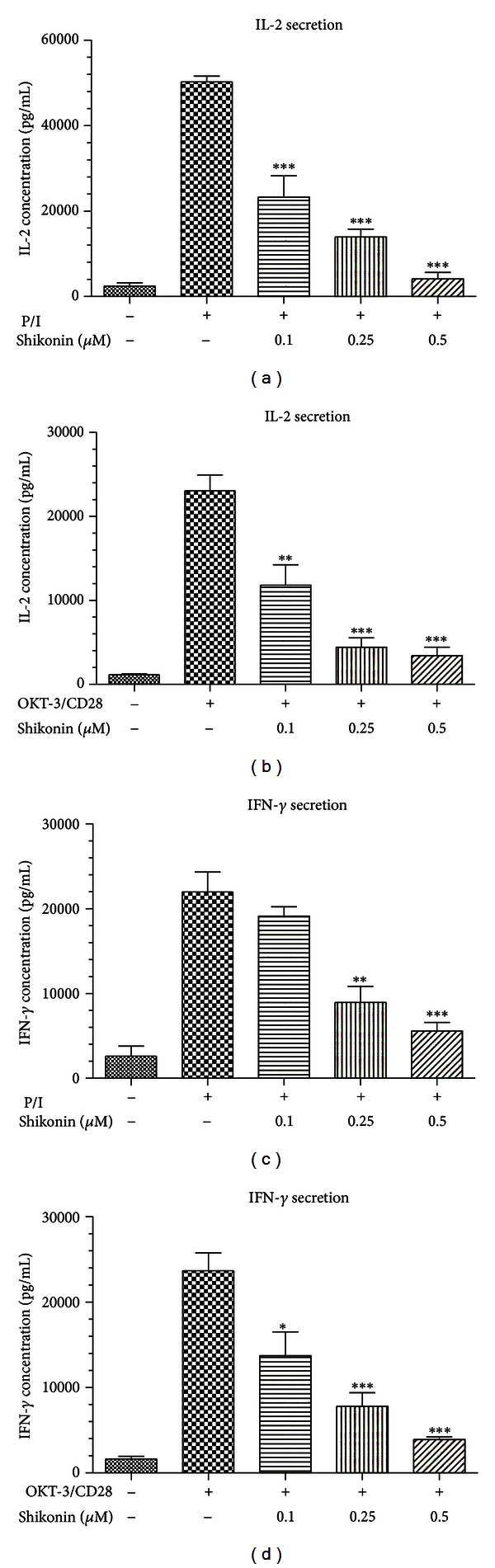
Effect of shikonin on inhibition of IL-2 (a and b) and IFN-*γ* secretion (c and d) in human T lymphocytes stimulated by PMA/ionomycin or OKT-3/CD28. The cells (10^5^/well) were firstly treated with shikonin for 2 h and then stimulated with PMA (20 ng/mL)/ionomycin (1 *μ*M) (P/I) or with the coated OKT-3 (5 *μ*g/mL)/CD28 (1 *μ*g/mL) (OKT-3/CD28) for 48 h. The level of IL-2 and IFN-*γ* in cell culture supernatants was determined by ELISA method. Data reported represent the mean ± SEM of three independent experiments. Significance of differences shows as *P < 0.05, **P < 0.01, ***P < 0.001.

T cell proliferation depends on cytokines secretion, especially IL-2 and IFN-*γ*. To evaluate whether the inhibitory effect of shikonin on human T-cell proliferation was mediated by inhibition of IL-2 and IFN-*γ* secretion, we examined the effect of shikonin on IL-2 and IFN-*γ* secretion. As shown in Figure 2, IL-2 and IFN-*γ* were significantly secreted in the cells evoked by PMA/ionomycin, while this increased secretion could be abolished by treatment of shikonin in a dose-dependent manner.

### 3.3. Shikonin Arrests Cell Cycle of the Human T Lymphocytes

**Figure 3:**
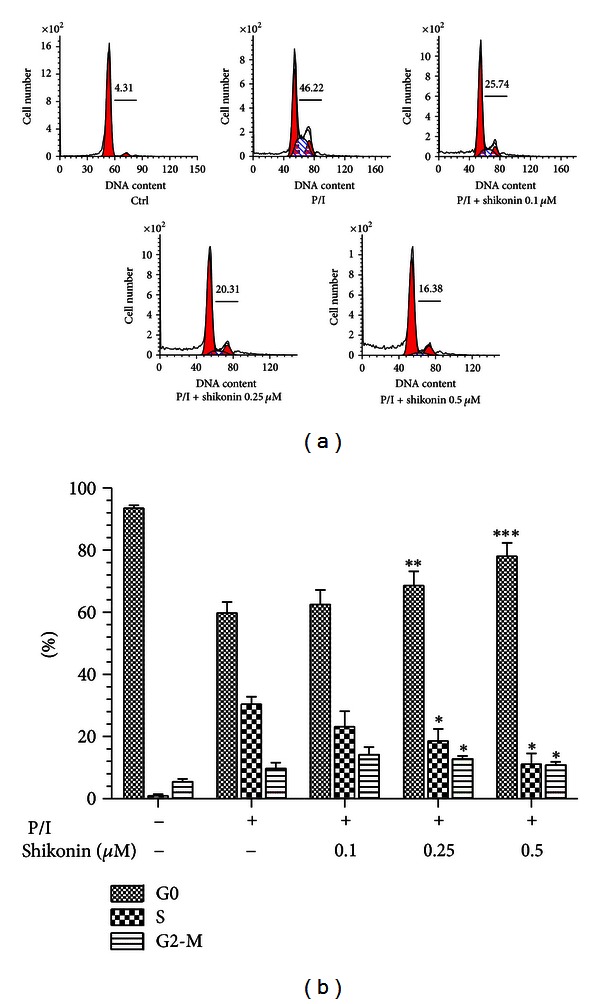
Effect of shikonin on the cell cycle of human T lymphocytes stimulated by PMA/ionomycin. Human T cells (10^6^) were pretreated with shikonin for 2 h then cultured with or without PMA (20 ng/mL)/ionomycin (1 *μ*M) (P/I) for 72 h. The cell populations were measured by flow cytometry, and total percentages of the cells entering the S and G2/M phases of the cell cycle were indicated. Data are a representative experiment out of three independent experiments with similar results.

To further elucidate underlying mechanism of shikonin on suppression of T lymphocyte proliferation, IL-2 and IFN-*γ* secretion, nuclear DNA of the cells was stained by propidium iodide, and then the cell cycle was analyzed by using flow cytometry. As shown in Figure 3, the cells remained largely in the G0/G1 phase in the resting T cells, while after stimulated with PMA/ionomycin, the cells were well activated and progressed through S, G2, and M phases of the cell cycle. However, when the cells were pretreated with 0.25 or 0.5 *μ*M of shikonin, cycling of those cells was blocked in the G0/G1 phase compared to the nonpretreated cells, and the entry of cells into the S phase of cell cycle was significantly prevented.

### 3.4. Shikonin Inhibits CD69, CD25, and CD71 Expression on Human T Lymphocytes

**Figure 4:**
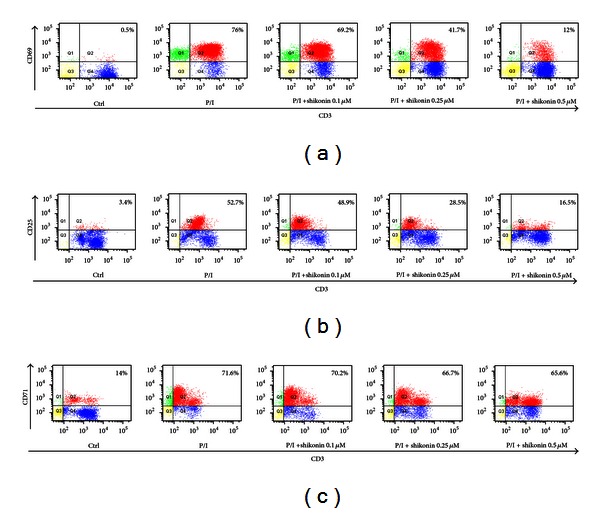
Effect of shikonin on CD25, CD69, and CD71 expression on human T lymphocytes. Human T lymphocytes (10^6^) were pretreated with shikonin for 2 h and then stimulated by PMA (20 ng/mL)/ionomycin (1 *μ*M) (P/I) for 48 h or 72 h, respectively. The cells were double stained with PE-CD3 and FITC-CD69 (a), PE-CD3 and FITC-CD25 (b), PE-CD3 or FITC-CD71 (c) antibodies and then analyzed by flow cytometry. The unstimulated cells were served as negative control. Values represent percentages of the double stained cells.

The entry of T cells into the cell cycle and their subsequent progression through G1 phase is accompanied by activation of numerous cellular events including expression of the surface markers of CD69, CD25, and CD71. Our results demonstrated that stimulation with PMA/ionomycin in human T lymphocytes induced expression of CD25, CD69, and CD71 up to 76.0%, 52.7%, and 71.6%, respectively, while shikonin produced suppression of CD69 and CD25 expression to 12.0% and 16.5%. However, shikonin slightly suppressed CD71 expression to 65.6% (see Figure 4).

### 3.5. Shikonin Inhibits NF-*κ*B Signaling of Human T Lymphocytes

**Figure 5:**
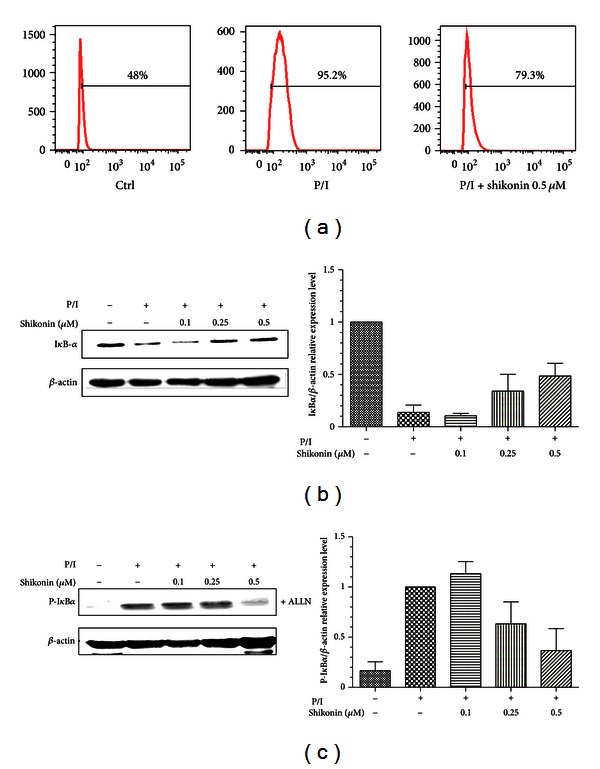
Effect of shikonin on inhibition of nuclear translocation of NF-*κ*B subunit p65 (a), degradation and phosphorylation of I*κ*B-*α* in human T lymphocytes stimulated by PMA/ionomycin (b and c). For analysis of the intercellular NF-*κ*B expression, cells were incubated with shikonin for 60 min, and then fixed immediately by cytofix buffer after costimulation by PMA (20 ng/mL)/ionomycin (1 *μ*M) for 120 min, stained with NF-*κ*B antibody for 60 min avoiding light, and then analyzed by flow cytometry. The unstimulated cells were served as negative control (a). For detection of I*κ*B-*α*, cells were incubated with or without shikonin for 60 min (b); for detection of pI*κ*B-*α*, the human T lymphocytes were pretreated with or without shikonin and 100 *μ*M ALLN for 60 min (c) and then stimulated with PMA (20 ng/mL)/ionomycin (1 *μ*M) (P/I) at 37°C for 60 min. The whole-cell lysates were prepared, and the proteins were analyzed by Western blotting using antibodies against I*κ*B-*α* and P-I*κ*B-*α*. Data are representative of three independent experiments.

CD25 appears to be regulated at the transcriptional level by CD28 through NF-*κ*B signaling which is mainly regulated by the classical NF-*κ*B p50–p65 complexes [19], and then we further examined whether expression of NF-*κ*B signaling in the activated human T lymphocytes could be inhibited by shikonin. The data were analyzed by flow cytometry, and the results indicate that the level of NF-*κ*B nuclear expression in the cells could be significantly elevated by stimulation of PMA/ionomycin. As we expected, the level of NF-*κ*B expression was obviously decreased by treatment of shikonin at 0.5 *μ*M (Figure 5(a)). Furthermore, nuclear translocation of p65 is preceded by phosphorylation and degradation of I*κ*B-*α*. To determine whether inhibition of NF-*κ*B activation by shikonin was due to inhibition of I*κ*B*α* degradation, we examined the level of degradation and phosphorylation of I*κ*B-*α* in human T lymphocytes stimulated by PMA/ionomycin in the absence and presence of shikonin. Tha results showed that PMA/ionomycin induced degradation of I*κ*B-*α*, while shikonin markedly suppressed this degradation in a dose-dependent manner (Figure 5(b)). To further determine if the inhibitory effect of shikonin on I*κ*B*α* degradation induced by PMA/ionomycin was associated with inhibition of I*κ*B*α* phosphorylation, we used the proteasome inhibitor N-acetyl-leucyl-leucyl-norleucinal (ALLN) to block degradation of I*κ*B*α* in the experiment, as results showed that I*κ*B*α* phosphorylation was strongly suppressed by shikonin (Figure 5(c)).

### 3.6. Shikonin Directly Suppresses IKK*β* Activity

**Figure 6:**
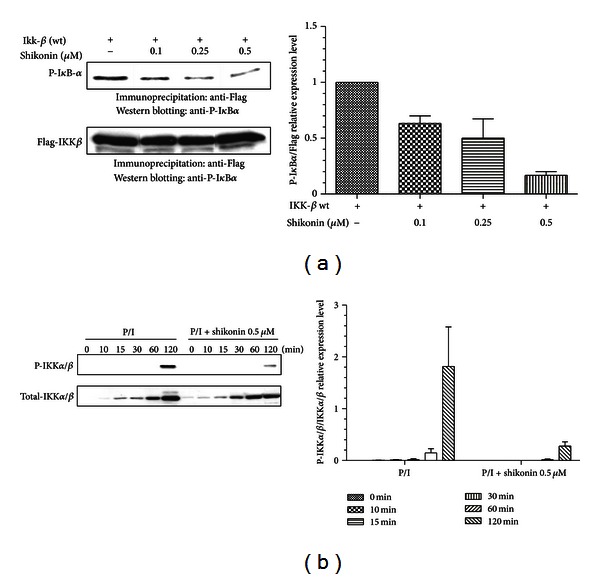
Effect of shikonin on inhibition of IKK-*β* activity (a) and IKK-*α*/*β* phosphorylation (b). Human HEK293 cells transfected with FLAG-IKK-*β* (wt) plasmid were immunoprecipitated (IP) with anti-Flag antibody, and the IP Flag-IKK-*β* was incubated with GST-I*κ*B*α* substrate and ATP in the presence or absence of 100 *μ*M shikonin. IKK-*β* kinase activity was determined by the level of phosphorylated GST-I*κ*B*α* using antibody against p-I*κ*B*α* (a). The human T lymphocytes were pretreated with shikonin at 37°C for 60 min and then stimulated with PMA (20 ng/mL)/ionomycin (1 *μ*M) (P/I) at 37°C for different time points. The whole-cell lysates were prepared, and proteins were analyzed by Western blotting using antibodies against IKK*α*/*β* and the phosphorylated form of IKK*α*/*β*. Data are representative of three independent experiments.

IKK is responsible for the phosphorylation and degradation of I*κ*B-*α*, while activation of IKK-*β*, rather than IKK-*α*, participates in the classical signaling pathway by which the pro-inflammatory stimuli induce NF-*κ*B activation through the phosphorylation of I*κ*B-*α*. In the current study we found that shikonin significantly inhibited phosphorylation and degradation of I*κ*B-*α* in human lymphocytes, and therefore we further examined if shikonin could directly inhibit the IKK-*β* activity. The results clearly showed that shikonin at 0.25 *μ*M and 0.5 *μ*M significantly suppressed the activity of IKK*β* kinase, probably via direct interactions (Figure 6(a)). We further determined whether shikonin could reduce the phosphorylation of IKK*β* induced by PMA/ionomycin. The human T lymphocytes were pretreated with shikonin and then exposed to PMA/ionomycin for various time periods. Subsequently, the IKK*α*/*β* phosphorylation in total cell extracts was determined by Western blot analysis. The results shown in Figure 6(b) indicated that PMA/ionomycin induced IKK*α*/*β* phosphorylation at 120 min, while shikonin concentration significantly prevented phosphorylation of IKK*α*/*β* at 0.5 *μ*M.

### 3.7. Shikonin Inhibits Phosphorylation of JNK

**Figure 7:**
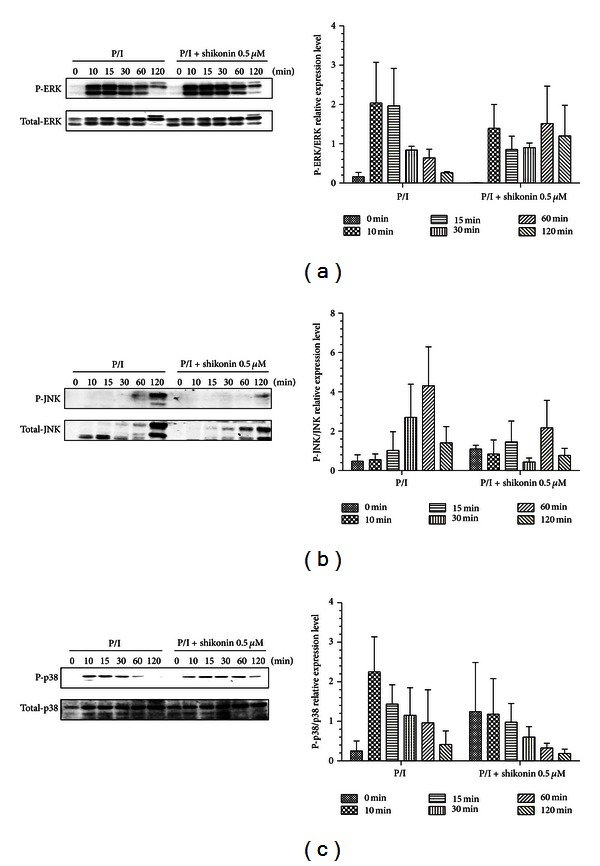
Effect of shikonin on MAPK phosphorylation stimulated by PMA/ionomycin. The human T lymphocytes were pretreated with shikonin at 37°C for 60 min and then costimulated with PMA (20 ng/mL)/ionomycin (1 *μ*M) (P/I) at 37°C for different time points. The whole-cell lysates were prepared, and proteins were analyzed by Western blotting using antibodies against ERK, JNK, and p38 and the phosphorylated forms of ERK, JNK, and p38 (a, b, and c). Data are representative of three independent experiments.

MAPKs composed of ERK, JNK, and p38 kinase serve as the most ancient signal transductional pathway involving T-cell activation [20] and IL-2 expression [21]. So, we further examined the effect of shikonin on the MAPKs signaling in human T lymphocytes. Total cellular extractions of the cells were prepared, and the signal transduction protein was measured by Western blotting. The results showed that shikonin could obviously suppress JNK phosphorylation but has no influences on ERK and p38 phosphorylation (Figure 7).

## 4. Discussion

Previous studies showed that shikonin has diverse pharmacological properties such as antiinflammation and anti-cancer. It could also inhibit the transcriptional activity of cyclooxygenase-2, TNF-*α* promoters [22], nitric oxide synthase induction, NF-*κ*B nuclear translocation, as well as the binding of NF-*κ*B to DNA in the RAW264.7 cells, and peritoneal macrophages isolated from Balb/C mice as well [7, 23]. It was reported that shikonin induced apoptosis of macrophages via inhibition of their proteasome as well [24]. Moreover, it has been demonstrated that shikonin effectively suppressed maturation of bone marrow-derived dendritic cells (BM-DC) induced by ovalbumin (OVA) and thymic stromal lymphopoietin (TSLP) *in vitro* [2]. We found that investigation of anti-inflammatory effect of shikonin mainly focused on the macrophage. Physiologically, T cell is another dominant cell population for mediating immune and inflammatory responses in humans and plays the key role in the secretion of cytokines as well as induction of inflammatory diseases; however, there is no report regarding the action of shikonin or its derivatives on T cells. In the current study, it is the first time to demonstrate the inhibitory property of shikonin on human T lymphocytes, namely, significant suppressions on the T-cell proliferation, IL-2 and IFN-*γ* secretion, cell cycle arrest and cell surface marker activation, through inhibition on NF-*κ*B signaling, and JNK phosphorylation via direct abrogate IKK*β* activity.

Activation and clonal expansion of T cells is the central event in the generation of immune and inflammatory responses. Productive T-cell activation depends on the essential signal (signal 1) provided by peptide/MHC complex and additional signal (signal 2) provided by CD28 [25]. Costimulation of CD28 and the immobilized anti-CD3 antibody can dramatically augment T-cell responses showing proliferation and cytokine secretion [26]. Moreover, PMA, one of phorbol esters and diacyl glycerol analogs, could stimulate PKC*θ* activity, while ionomycin, one of calcium ionophores, results in an increase at the intracellular calcium level due to the higher extracellular calcium concentration. PMA/ionomycin can lead to T-cell activation through bypass surface TCR engagement and cross-linking requirements and directly activates intracellular signaling pathways [27]. Thus, in our current studies both OKT-3/CD28 and PMA/ionomycin were employed to elicit T-cell activation responses, which may fit to the immune and inflammatory responses in clinic as well as the translational research for developing a candidate anti-inflammatory drug. We found that shikonin significantly inhibited T cell proliferation, IL-2 and IFN-*γ* secretion induced by either PMA/ionomycin or OKT-3/CD28, indicating that shikonin may have a potency of inhibiting PKC*θ* or its downstream. After being calculated, we found that shikonin inhibited T-cell proliferation with IC_50_ values of 2.4 *μ*g/mL. Although the concentration is relatively higher than cyclosporine A (IC_50_ = 10 ng/mL) [28], a classical immunosuppressive drug, the immune-suppressive effect of shikonin on T-cell proliferation is better than other compounds derived from plant medicine, such as Suberosin and Pseudolaric acid B, of which effective concentration is 100 *μ*M and 10 *μ*M, respectively [29, 30].

IL-2 transcription and secretion promote T-cell cycle progression and effector functions in the activated T cells [31]; hence, we further investigated the effect of shikonin on the cell cycle. Resting T cells are mainly arrested in G0 phase, while the cells can enter into the cell cycle to proliferate when they are challenged by antigen or mitogen [18, 32]. In the present study, we found that shikonin treatment could prevent cells from entering the phases of cell cycle, implying that shikonin-mediated cell cycle arrest might further contribute to the inhibition of T-cell proliferation, production of the growth factors of T cells including IL-2 and IFN-*γ* secretion [33, 34]. As there was no cytotoxicity of shikonin on human T lymphocytes at 0.5 *μ*M, it can be concluded that the immunosuppressive effect of shikonin on human T lymphocytes is resulted from its pharmacological inhibitory property.

To further elucidate the underlying molecular mechanisms of shikonin on T-cell activation, we further investigated its action on T-cell activation markers, including CD25 (IL-2*α* receptor), CD69, and CD71 [35]. CD25 can mediate full expression of immune responses through interacting with IL-2 and its receptors, triggering cellular proliferation, and culminating in the emergence of effector T cells [36]. In general, CD25 is regulated by CD28 at transcriptional level through NF-*κ*B signaling and highly expressed during T-cell activation [37–39]. Meanwhile CD69 is the earliest T-cell activation, while CD71 is the latest T cell activation marker [40]. All of these markers participate in T-cell proliferation, and levels of these markers correlate with the degree of immune responses. Results in the current study showed that shikonin could significantly suppress CD25 and CD69 expression but slightly influence CD71 expression. Considering the close correlations between CD25 expression and NF-*κ*B signaling we further proposed that shikonin might inhibit T-cell activation by blocking NF-*κ*B signaling. Moreover, NF-*κ*B regulates IL-2 production and T-cell proliferation. Consequently, we further performed experiments to clarify the effect of shikonin on NF-*κ*B signaling pathway.

The constitutive activation of NF-*κ*B signaling is often associated with inflammatory and autoimmune conditions [41]. Recently the strategies of regulation or inhibition of NF-*κ*B signaling has been deeply investigated for drug discovery, such as suppression of 26S proteasome and interfere with the binding of NF-*κ*B to DNA. Inhibition on 26S proteasome has been evident of one of the attractive targets for suppressing NF-*κ*B activation, as it could inhibit I*κ*B*α* phosphorylation and degradation, and NF-*κ*B nuclear translocation as well. However, the proteasome is involved in the degradation of all polyubiquitinated proteins; thus it is difficult to find the most specific inhibitors on the enzymes like E3 ubiquitin ligases and E3 ubiquitin-conjugating enzymes, which are responsible for the phosphorylation-dependent polyubiquitination of I*κ*Bs [13]. Considering those complexities above, searching for the inhibitors on the IKK activity may offer the most effective and selective strategy for suppression of NF-*κ*B activation [13]. Our present data demonstrated that shikonin could significantly suppress NF-*κ*B signaling pathway through direct suppression of the IKK*β* activity, indicating prevention of the NF-*κ*B nuclear translocation, and I*κ*B*α* phosphorylation and degradation, IKK*α*/*β* phosphorylation.

MAPK cascades play important role in regulating IL-2 expression [21], and inhibition of ERK or p38 phosphorylation has been proven to prevent IL-2 expression [42, 43], which indicates that both of them are essential for T-cell activation. Moreover, JNK could phosphorylate c-jun, a member of the AP-1 transcriptional factor family which can generate T-cell activation and is involved in gene transcriptional activity of IL-2 [44, 45]. Thus, we investigated the effect of shikonin on MAPK signaling, and the data showed that shikonin inhibited JNK phosphorylation without influence on the phosphorylation of ERK and p38. JNK pathway seems to play multiple roles in T-cell immune responses, as it can be activated in T cells by stimulation, modulation of cytokine secretion, and cell proliferation [46, 47]. Taken together, the inhibitory effect of shikonin on human T lymphocytes may mainly result from suppression of IKK*β* activity in the cells.

## 5. Conclusion

In summary, the current studies have firstly demonstrated immunosuppressive effect of shikonin on human T lymphocytes through suppression of cell activation, while the major molecular mechanisms are involved in inhibition of CD25, CD69 expression, cell cycle, NF-*κ*B and JNK signaling, and IKK*β* activity. Based on the suppressive effect of shikonin on human T cells, shikonin may have significant potentials to be investigated as a lead compound for the design and development of a new immunosuppressant for preventing graft rejection and treating autoimmune diseases.
